# Features of the spectrum of immune markers in patients with juvenile depression with clinically high risk of psychosis

**DOI:** 10.1192/j.eurpsy.2024.583

**Published:** 2024-08-27

**Authors:** S. A. Zozulya, M. A. Omelchenko, I. N. Otman, Z. V. Sarmanova, V. V. Migalina, V. G. Kaleda, T. P. Klyushnik

**Affiliations:** Mental Health Research Centre, Moscow, Russian Federation

## Abstract

**Introduction:**

Identification of biomarkers associated with the risk of psychosis manifestation in juvenile patients with depression may contribute to a better understanding of the pathogenesis of mental disorders and early diagnosis.

**Objectives:**

To determine the level of pro-inflammatory and anti-inflammatory cytokines and other inflammatory indicators in the plasma of juvenile patients with depression and clinically high risk of psychosis, and to study the correlation of these markers with the severity of psychopathologic symptoms.

**Methods:**

80 young men aged 16-24 years with the first depressive episode (F32.1-2, F32.38, F32.8) were examined. Based on the severity of attenuated psychotic symptoms (APS) in the structure of depression according to the SOPS scale, all patients were divided into two groups - with clinically high risk of psychosis (n=58) and with depression without APS (n=22). The HDRS-21 and SANS scales were also used for psychometric assessment. Serum level of cytokines TNF-α, IL-6, IL-8, IL-10, TNF-α/IL-6 ratio, TNF-α/IL-10 ratio, leukocyte elastase (LE) and α1-proteinase inhibitor (α1-PI) activity, C-reactive protein (CRP) concentration, and the level of autoantibodies to S-100B protein were determined.

**Results:**

Both groups of patients showed a high level of inflammation assessed by LE and α1-PI activity (p>0.05). Significantly higher level of IL-6 (p=0.03), CRP concentration (p=0.026) and TNF-α/IL-10 ratio (p=0.032) were found in patients with clinically high risk of psychosis. This group was also characterised by high level of autoantibodies to the S-100B protein compared to patients with depression without APS (p=0.048).

In the high clinical risk group, correlations were found between the SOPS positive subscale score and the level of TNF-α (R=0.32, p=0.017), IL-8 (R=-0.3, p=0.034), TNF-α/IL-6 ratio (R=0.30, p=0.021) and TNF-α/IL-10 ratio (R=0.32, p=0.014). The SOPS negative subscale score correlated with CRP concentration (R=0.3, p=0.043). The SOPS total score correlated with TNF-α/IL-10 ratio (R=0.31, p=0.021). In this group of patients, the level of IL-10 was found to correlate with the duration of the disease (R=0.48, p<0.001). In patients with depression without APS, the level of IL-6 was correlated with the severity of depression according to the HDRS scale, and the level of TNF-α was associated with the duration of the depressive episode (R=0.51, p=0.029).

**Conclusions:**

The obtained results confirm the involvement of inflammation in the development of juvenile depression. Qualitative and quantitative characteristics of the spectrum of immune markers and the cytokine profile, and correlations with the severity of psychopathologic symptoms were revealed in patients with clinically high risk of psychosis.

**Disclosure of Interest:**

None Declared

